# AI‐Driven Synthesis in Medicinal Chemistry: Integrating Large Language Models, Robotic Automation, and Sustainability Metrics to Accelerate Drug Discovery

**DOI:** 10.1002/med.70074

**Published:** 2026-06-17

**Authors:** Amit Gangwal, Antonio Lavecchia

**Affiliations:** ^1^ School of Pharmacy and Technology Management SVKM NMIMS Global University Dhule Maharashtra India; ^2^ “Drug Discovery” Laboratory, Department of Pharmacy University of Naples Federico II Italy

**Keywords:** artificial intelligence, autonomous synthesis, green chemistry, large language models, medicinal chemistry, retrosynthesis

## Abstract

Artificial intelligence (AI) is transforming synthetic chemistry from task‐specific predictors into integrated platforms that unify retrosynthesis, reaction optimization, and closed‐loop robotic automation. This review highlights how AI‐assisted planning and robotic execution shorten cycle times, reduce step counts, and improve route sustainability in medicinal chemistry. Recent advances, including large language models (LLMs), template‐free retrosynthesis, and Bayesian optimization, are evaluated alongside key limitations in dataset quality, reproducibility, and deployment costs. To ensure translational relevance, reproducible benchmarks such as step count, time‐to‐in vitro, and green metrics (E‐factor, process mass intensity) are emphasized. This review proposes a hierarchical framework structured across three interconnected levels: cognitive planning, physical execution, and translational evaluation. Within this structure, key elements include LLM‐based synthesis planning, robotic and closed‐loop execution, interpretable decision‐making, sustainability‐by‐design, advanced reaction optimization, and multi‐objective retrosynthesis. Together, these components provide a conceptual basis for integrating digital intelligence with physical experimentation. By embedding green chemistry principles and regulatory awareness, AI is increasingly positioned not only as a predictive tool but also as an assistive collaborator supporting decision‐making in medicinal chemistry workflows. The convergence of AI, robotics, and sustainability metrics highlights an emerging transition; however, realizing a future where every experiment reliably feeds back into autonomous learning loops requires overcoming significant current barriers in data standardization and hardware interoperability.

**Abbreviations**
3D: three‐dimensional
ADME: absorption, distribution, metabolism, and excretion
API: active pharmaceutical ingredient
BERT: bidirectional encoder representations from transformers
BO: Bayesian optimization
CASP: computer‐aided synthesis planning
DFT: density functional theory
DL: deep learning
DMTA: Design–Make–Test–Analyze
DMTL: design–make–test–learn
EDBO+: experimental design via BO
GA: genetic algorithms
GMP: good manufacturing practice
GNN: graph neural networks
GP: Gaussian processes
HITL: human‐in‐the‐loop
HPLC: high‐performance liquid chromatography
IP: intellectual property
LLM: large language models
MCTS: Monte Carlo Tree Search
ML: machine learning
MOF: metal–organic frameworks
MOO: multi‐objective optimization
MORL: multi‐objective reinforcement learning
MS: mass spectrometry
NLP: natural language processing
NMR: nuclear magnetic resonance
NSGA‐II: Non‐dominated Sorting Genetic Algorithm II
ORD: open reaction database
PAT: process analytical technology
QML: quantum machine learning
R&D: research and development
SAR: structure–activity relationship
SCROP: self‐corrected transformer
SDL: self‐driving laboratories
SHAP: SHapley Additive exPlanations
THE: high‐throughput experimentation
TNIK: TRAF2‐ and NCK‐interacting kinase
XAI: Explainable AI
XDL: extensible data language

## 1 Introduction

Artificial intelligence (AI) is increasingly influencing synthetic chemistry and reshaping several of its core paradigms. This review critically evaluates how AI‐driven synthesis technologies, including autonomous planning systems and robotic execution platforms, enhance capabilities in medicinal chemistry, supporting the design and planning of complex drug molecules with improved efficiency, safety, and environmental sustainability. Importantly, beyond providing a descriptive overview, this review critically examines the real‐world applicability, limitations, and translational readiness of current AI‐driven synthesis technologies, explicitly distinguishing between experimentally validated achievements, proof‐of‐concept systems, and forward‐looking expectations. Unlike traditional reviews that focus on individual computational tools, we emphasize the emerging integration of AI with laboratory automation, interpretable decision‐making, and sustainability‐oriented design. This integration reflects a shift from predictive assistance toward more coordinated systems that support medicinal chemistry decision‐making and drug discovery workflows.

Early AI tools showed strong performance in discrete tasks such as reaction prediction, retrosynthetic analysis, molecular design, and property optimization. The field is now progressing toward end‐to‐end frameworks that connect digital planning with physical execution, enabling streamlined drug discovery pipelines. This evolution is driven by advances in deep learning (DL), the expansion of reaction databases, and the emergence of large language models (LLMs), which show potential for capturing patterns related to structure–activity relationships (SAR) and synthetic accessibility, although their predictions remain dependent on training data and require experimental validation [1, 2].

Natural language processing (NLP) provides the conceptual foundation for these chemical LLMs. Transformer architectures, such as BERT (Bidirectional Encoder Representations from Transformers) and modern generative models like GPT‐4, encode and manipulate molecular representations (SMILES, SELFIES), enabling interpretation, translation, and generation of chemical structures [3]. When integrated with computer‐aided synthesis planning (CASP) tools, LLMs can propose candidate disconnections, assess synthetic feasibility, and suggest multi‐step routes; however, these capabilities remain dependent on integration with specialized tools and require experimental validation to ensure chemical correctness.

Self‐driving laboratories (SDLs) integrate planning algorithms, robotics, and real‐time analytics, exemplified by platforms such as ChemCrow [4], Sonidegib optimization, and closed‐loop Bayesian approaches [5, 6, 7, 8]. These systems accelerate medicinal chemistry workflows, enabling rapid identification of viable synthetic routes, multi‐objective optimization (MOO) of molecular properties, and integration of sustainability considerations.

Despite these successes, several open challenges remain, including the limited generalization of models to out‐of‐distribution substrates, the need for integrated evaluation of reaction conditions, and the alignment of AI‐generated routes with medicinal chemistry priorities such as scaffold diversity and late‐stage functionalization.

To provide a structured perspective, we organize the current landscape of AI‐driven synthesis into a three‐tiered hierarchical framework based on functional roles within synthesis workflows. This framework distinguishes between decision‐making and retrosynthetic reasoning, their experimental realization through automated systems, and the criteria required for real‐world applicability: (1) cognitive planning, encompassing LLM‐driven reasoning and multi‐objective retrosynthetic design; (2) physical execution, translating digital plans into reality through robotic closed‐loop systems and reaction optimization algorithms; and (3) translational evaluation, ensuring applicability through interpretability, sustainability, and scalability considerations. Rather than treating objectives such as green chemistry or condition optimization as isolated pillars, this hierarchy embeds them as cross‐cutting criteria guiding planning and execution. Accordingly, sustainability, reaction optimization, and multi‐objective design are treated as evaluation dimensions that influence decisions across all levels.

This framework provides a structured perspective for merging digital intelligence with physical experimentation, addressing both the efficiency and strategic requirements of modern drug discovery. Key challenges persist: template‐free retrosynthesis models, while flexible, often generate chemically implausible disconnections when applied to out‐of‐distribution substrates, with error rates exceeding 50% in some cases [9]. Similarly, models trained on existing reaction yield data have limited generalization for predicting high‐yield outcomes due to dataset biases toward low‐yield reactions [10]. This review addresses these limitations by emphasizing hybrid human–AI strategies, interpretable architectures, and prospective experimental validation as key requirements for pharmaceutical adoption.

## 2 AI‐Driven Synthesis Planning and Execution

### 2.1 Reaction Prediction and Retrosynthesis

Accurately predicting reaction outcomes, yields, selectivity, and kinetics remains a central challenge in synthetic chemistry, especially for designing and optimizing bioactive small molecules. Traditionally, this relied on time‐intensive trial‐and‐error and chemists' intuition [11]. Recent advances in AI have established a data‐driven foundation for chemical reasoning, where models learn from large reaction datasets rather than heuristic rules. Supervised learning, neural networks, decision trees, ensemble models, and graph neural networks (GNNs) now analyze millions of curated reactions to predict optimal conditions, improve selectivity, and propose feasible synthetic strategies [12]. These approaches enhance reagent and catalyst selection, reduce side‐product formation, and adapt to emerging paradigms such as flow chemistry and continuous manufacturing. Mechanistically‐annotated large‐scale datasets (e.g., mech‐USPTO‐31K) further improve model robustness for yield and condition prediction [13]. By integrating mechanistic and physicochemical constraints, AI‐based reaction prediction can support more reproducible and scalable synthesis design, although reproducibility depends strongly on data quality and experimental validation.

Building upon this predictive layer, retrosynthesis models extend AI's role from reaction‐level forecasting to multistep route design in medicinal chemistry [14]. Template‐free methods can propose plausible transformations, though they frequently generate chemically invalid or impractical suggestions, particularly outside training distributions. Approaches such as Retro‐Score perform full retrosynthetic analyses to evaluate synthesizability, accelerating lead optimization and reducing human bias [15, 16, 17].

While benchmark accuracies are high, multi‐step planning remains limited by error propagation and lack of interpretability [18]. To address these issues, recent models incorporate mechanistic filters, quantum descriptors, and brief human‐in‐the‐loop (HITL) validation. For example, the Self‐Corrected Transformer achieved 59.0% top‐1 accuracy on the USPTO‐50k benchmark while reducing invalid SMILES from 12.1% to 0.7% [19]. Grammar‐aware SMILES representations also improve syntactic validity and MaxFrag accuracy [20].

Hybrid frameworks that combine data‐driven prediction with symbolic reasoning, embedding chemical validity checks (valence, atom mapping, solvent/catalyst compatibility) [21], quantum‐augmented scoring (e.g., ml‐QM‐GNN) [22], and experience‐guided Monte Carlo Tree Search (MCTS) [23] are emerging as the most reliable strategy for scalable retrosynthetic planning. Despite this progress, benchmarking still reveals persistent limitations in navigating broad chemical spaces for multi‐step synthesis, as exemplified by AiZynthFinder [24]. These predictive frameworks now underpin the next phase of automated synthesis, where LLMs and agentic systems extend AI reasoning beyond reaction‐level prediction.

### 2.2 LLMs and Agentic Frameworks for Synthesis Planning

Building on this conceptual foundation, LLMs extend chemical AI beyond reaction prediction toward reasoning, planning, and autonomous decision support. LLMs such as GPT‐4 and chemistry‐adapted variants are now explored as integrative components of synthesis planning and decision‐making, interpreting SMILES, extracting structured chemical knowledge, and proposing multi‐step strategies when connected to specialized toolchains. This paradigm shifts computational chemistry from static prediction to adaptive decision support, accelerating hypothesis refinement and route prioritization, while chemists retain control over mechanistic judgment and safety‐critical execution [25].

When strictly constrained by external validation tools and expert‐designed prompts, LLMs can serve as assistive interfaces to help draft candidate synthetic plans; however, their outputs are not inherently reliable and must be constrained by validation tools, mechanistic filters, and expert supervision. Rather than acting as independent reasoning engines, they rely entirely on integration with established predictive models and heuristic filters to prevent the generation of chemically implausible routes. This approach aligns closely with medicinal chemistry practice, requiring a balance between synthetic feasibility, absorption, distribution, metabolism, excretion, and toxicity (ADMET) optimization, and structure‐based design under continuous data evolution. Structured validation pipelines, such as VALID‐Mol [26], markedly improve the fraction of chemically valid outputs (from ~3% to ~83%), highlighting the importance of combining generative LLM reasoning with expert‐guided filtering [4, 27].

Chemistry‐specific agents, such as ChemCrow [4] and community adaptations of Auto‐GPT [28], illustrate early implementations of this paradigm, serving as orchestration layers that combine LLM reasoning with domain‐specific tools. ChemCrow, for instance, can take a SMILES input, query chemical databases, propose retrosynthetic pathways, and refine suggestions based on synthetic feasibility, reagent availability, and cost‐efficiency [4]. These systems currently perform best in well‐defined problem spaces; robust performance in complex, high‐throughput campaigns remains under evaluation. Despite encouraging benchmark results, it is essential to distinguish between controlled computational evaluations and real‐world deployment scenarios, where reproducibility, experimental validation, and integration with laboratory workflows remain significant unresolved challenges.

Table 1 provides a comparative overview of representative agentic LLMs and chemical AI tools, reinforcing key differences in capabilities, scope, and integration with experimental platforms. These tools represent concrete implementations of the generalized agentic workflow illustrated in Figure 1, translating conceptual reasoning steps into executable synthesis planning and decision‐making processes. Agentic LLMs support creativity by proposing unconventional but literature‐grounded hypotheses. For example, LLM‐assisted workflows can aggregate docking scores, kinase selectivity, and SAR trends into ranked hypotheses for kinase inhibitor design. Human oversight remains indispensable for mechanistic plausibility, stereochemical correctness, and risk‐benefit evaluation [4, 27].

**Table 1:** Comparative overview of LLMs for pharmaceutical‐chemistry applications.

Model	Size/params	Main capabilities	Benchmark/performance highlights	Medicinal chemistry application	Software availability	Validation in pharma
ChemCrow [4]	N/A (wrapper using GPT‐4 API)	Tool‐augmented retrosynthesis and planning	Autonomous synthesis of insect repellents and organocatalysts.	Lead compound generation; rapid synthetic prototyping (e.g., insect repellent synthesis)	Open source	Pending
Auto‐GPT (Chem)	Variable; benchmarks N/P	Orchestration of cheminformatics tools and autonomous workflow chaining	Workflow integration; less detail on pharma substances	Hypothesis→design→execution automation in early R&D	Open source (community forks)	No
Coscientist [29]	GPT‐4‐based (params N/P)	Autonomous experimental design and robotic execution	Autonomous design, plan, and execution of chemical reactions (including palladium‐catalyzed cross‐couplings)	Closed‐loop optimization of drug‐relevant reactions	Closed source	Pending
ChemLLM [30]	N/P (preprint)	Comprehensive chemical reasoning (ChemBench)	Achieves near GPT‐4‐level performance on chemical NLP and reasoning tasks.	Property prediction and synthesis planning in hit‐to‐lead	Open source	No
ChemDFM [31]	13 B	Instruction‐based chemical dialog and prediction	Pre‐trained on large chemical corpus ~34 B tokens; fine‐tuned ~2.7 M instructions.	Extraction of reaction conditions; medchem retrosynthesis	Open source	No
ChemFM [32]	~3 B	Property prediction and molecule generation	Outperforms ChemBERTa and PubChemBERT in low‐resource settings.	Low‐resource learning for ADMET and scaffold optimization	Research release	No
BatGPT‐Chem [33]	N/P	Multi‐task retrosynthesis and reaction prediction	Top‐performing in multi‐task synthesis planning (preprint).	Prioritization of synthetic routes for drug‐like molecules	Research release	No
ChemPrompt + GPT [34]	GPT‐4‐based (params N/P)	High‐accuracy literature parameter extraction	Extracted > 26,000 synthesis parameters with ~99% accuracy for MOFs.	Automated curation of synthesis datasets	Partial/conditional availability	No
ChemDataExtractor [35]	N/A	Chemical literature mining (NER + table parsing)	F1 scores above ~80% for reaction/property dataset construction.	Reaction/property dataset construction for ML‐assisted design	Open source	No
CLAIRify [36]	N/A	NLP → XDL robotic translation	Proof‐of‐concept for robotic execution of synthetic protocols.	Automated execution of synthetic protocols	Research/demo only	No

Abbreviations: N/A, not applicable; N/P, not publicly disclosed.

**Figure 1:**
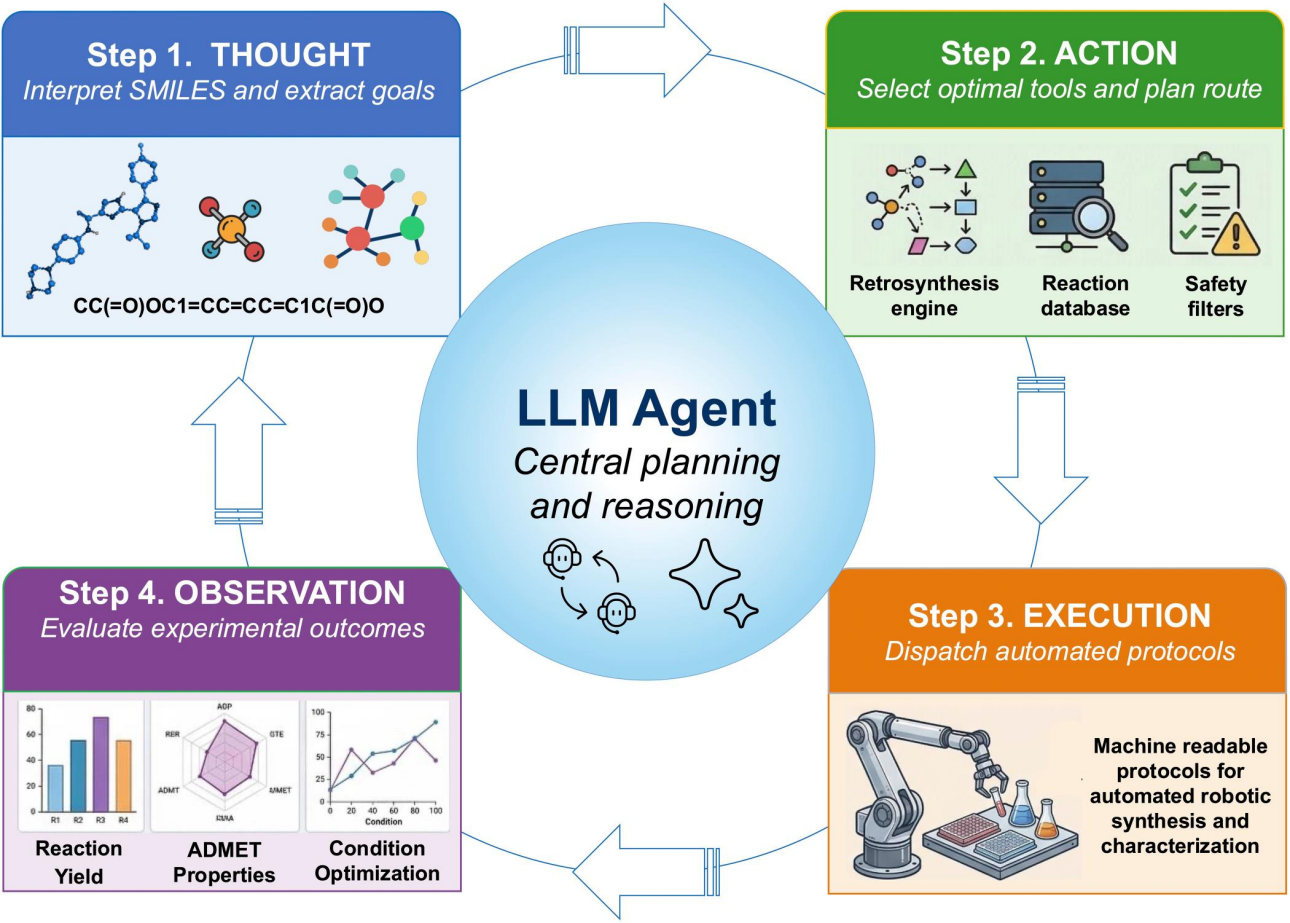
Workflow of an agentic LLM system for autonomous synthesis planning. The figure represents a generalized conceptual workflow rather than specific platform implementations. The cycle proceeds clockwise: (1) Thought—interpret input SMILES and extract objectives. (2) Action—select appropriate tools such as retrosynthesis engines or databases. (3) Execution—dispatch machine‐readable protocols to cloud labs or robotic platforms (e.g., RoboRXN). (4) Observation—evaluate yield, ADMET, and success metrics, then feed results back to Step 1. Representative implementations of these functionalities are discussed in the main text and summarized in Table 1. [Color figure can be viewed at wileyonlinelibrary.com]

Reflexive operation is a defining feature of agentic LLMs, allowing adaptation of synthetic plans in response to unexpected outcomes, resource constraints, or new computational analyses. This mirrors real‐world medicinal chemistry, where strategies evolve with ADMET readouts, scaffold challenges, and manufacturability barriers. Multi‐agent and domain‐tuned architectures extend this concept. For example, Coscientist, a GPT‐4‐driven multi‐agent system, autonomously proposes, plans, and executes complex experiments by integrating literature mining, code execution, and cloud‐lab interfacing [29]. Specialized chemical LLMs (ChemLLM, ChemDFM) achieve near GPT‐4‐level performance on synthesis planning, property prediction, and literature mining [30, 31]. ChemFM [32], a recent open‐source model trained on over 400 million molecules and 1.2 billion tokens, demonstrates robust generalization in low‐resource settings, outperforming established models such as ChemBERTa [37] and PubChemDeBERTa [38].

Despite encouraging benchmarks, practical deployment reveals recurrent limitations: hallucinations, edge‐case failures, stereochemical errors, and mechanistically implausible predictions [39, 40]. Robust uncertainty quantification, chemical validation, and prospective experimentation remain essential before LLM outputs can inform physical workflows. Training strategies and dataset engineering remain key performance levers. SMILES augmentation and improved decoding heuristics can match or exceed architectural upgrades [41]. Hybrid approaches that incorporate quantum‐mechanical or reaction‐mechanism priors further improve route quality, as exemplified by BatGPT‐Chem [33].

LLM‐assisted literature mining enables real‐time extraction of reaction conditions and experimental context. Prompt‐engineered GPT‐4 workflows such as ChemPrompt [34] extracted > 26,000 synthesis parameters from ~800 metal–organic frameworks with accuracies approaching 99%. ChemMiner [42] processed over 300 Suzuki reactions with F1 scores above 82%, greatly reducing manual curation. Tools such as ChemDataExtractor [35] extend this automation, populating machine learning (ML)‐ready datasets for synthesis planning and ADMET analysis.

In summary, LLM‐based reasoning establishes the cognitive interface between digital synthesis planning and robotic execution. Section 2.3 describes how these digital plans are operationalized through robotic automation, algorithmic optimization, and human–AI collaborative workflows. These capabilities establish the conceptual basis for translating AI‐driven planning into physical execution, as discussed in the following section.

### 2.3 Automated and Sustainable Synthesis Frameworks

This section describes how AI‐driven synthesis planning translates into physical execution through robotic infrastructures (Design–Make–Test–Analyze [DMTA] and SDL), algorithmic optimization strategies (Bayesian optimization [BO], active learning, MOO), human–AI collaborative workflows, and industrial case studies demonstrating quantifiable sustainability improvements.

#### 2.3.1 Hardware Infrastructures and Closed‐Loop Execution (DMTA and SDL)

The robotic translation of LLM‐generated procedures into executable protocols represents a major emerging capability. Figure 1 summarizes the core stages of an agentic workflow: input interpretation, tool selection, and execution, culminating in robotic platforms that operationalize autonomous synthesis planning. The schematic is intentionally presented as a generalized conceptual architecture, abstracting from specific implementations to emphasize functional components.

Text procedures can be translated into machine‐readable languages such as extensible data language (XDL), validated for syntax and safety, and executed on automated platforms [43, 44]. This synergy of algorithmic reasoning and robotic precision enables data‐rich, feedback‐driven experimentation. However, its impact on production‐scale chemistry remains dependent on system integration and validation across diverse reaction classes.

In practical applications, AI‐driven DMTA pipelines have already demonstrated reported acceleration of hit‐to‐validation timelines in selected case studies. For example, Liu et al. screened ~7500 compounds using neural networks and identified Abaucin, a narrow‐spectrum antibacterial, achieving in vivo efficacy in under 60 days and reducing the hit‐to‐validation timeline by over 75% compared with conventional workflows [45]. This case exemplifies how predictive modeling prioritizes synthetically tractable chemistries before physical synthesis, minimizing redundant experimentation and reagent waste. Table 2 contrasts traditional and AI‐driven synthesis workflows, exemplified by the Abaucin case study.

**Table 2:** Comparative performance of traditional and AI‐driven synthesis pipelines (Abaucin case study).

Metric	Traditional synthesis	AI‐driven synthesis (abaucin)
Total synthetic steps	~12–15 steps (template‐based medicinal chemistry routes) [46]	~3–4 steps from hit to in vitro validation [45]
Overall yield	~20%–30% overall (multiple protecting steps, purifications)	~40%–50%, enabled by optimized conditions and streamlined steps
SAR cycle time	≥ 6 months (synthesis, purification, testing)	≤ 1 month, thanks to AI‐suggested analogs and rapid screening
Lead‐to‐animal model	~12–18 months	~3–4 months via AI‐guided prioritization and synthesis
Selectivity scope	Broad, ex post facto evaluation on panels	Narrow‐spectrum focused on *A. baumannii* with minimal commensal impact^a^
Discovery timeline	Conventional pipeline	Weeks from in silico hit to in vitro validation [45]
Translation to efficacy	Traditional preclinical pipeline	In vivo efficacy testing in < 60 days

^a^ Abaucin exhibits narrow‐spectrum activity and low commensal toxicity in vitro and in vivo.

Further demonstrations of AI‐driven DMTA include microfluidic synthesis workflows, such as the synthesis of liver X receptor agonists, where 25 compounds were synthesized, 17 of which were active, with EC_50_ improvements up to 60‐fold and 12 confirmed upon batch resynthesis [28]. These results showcase the power of end‐to‐end integration between AI‐driven design, robotic synthesis, and real‐time validation.

Additional experimentally validated case studies further demonstrate the practical impact of AI‐assisted retrosynthesis. For example, a recent generative retrosynthesis framework based on template generation rather than reactant prediction enabled the design and experimental validation of a concise three‐step synthetic route for a structurally complex small molecule, improving upon previously reported routes requiring 5–9 steps [47]. This study highlights how AI‐driven retrosynthesis can directly translate into experimentally realizable and more efficient synthetic strategies, bridging the gap between computational design and laboratory execution.

SDLs extend this paradigm, enabling real‐time self‐optimization across multistep reactions by combining inline analytics (nuclear magnetic resonance [NMR], mass spectrometry, high‐performance liquid chromatography) with active feedback control [48]. The defining feature of SDLs is their closed‐loop architecture, as illustrated in Figure 2, where each stage is executed in a defined sequential order and organized within a circular feedback loop that continuously connects experimental execution to model updating.

**Figure 2:**
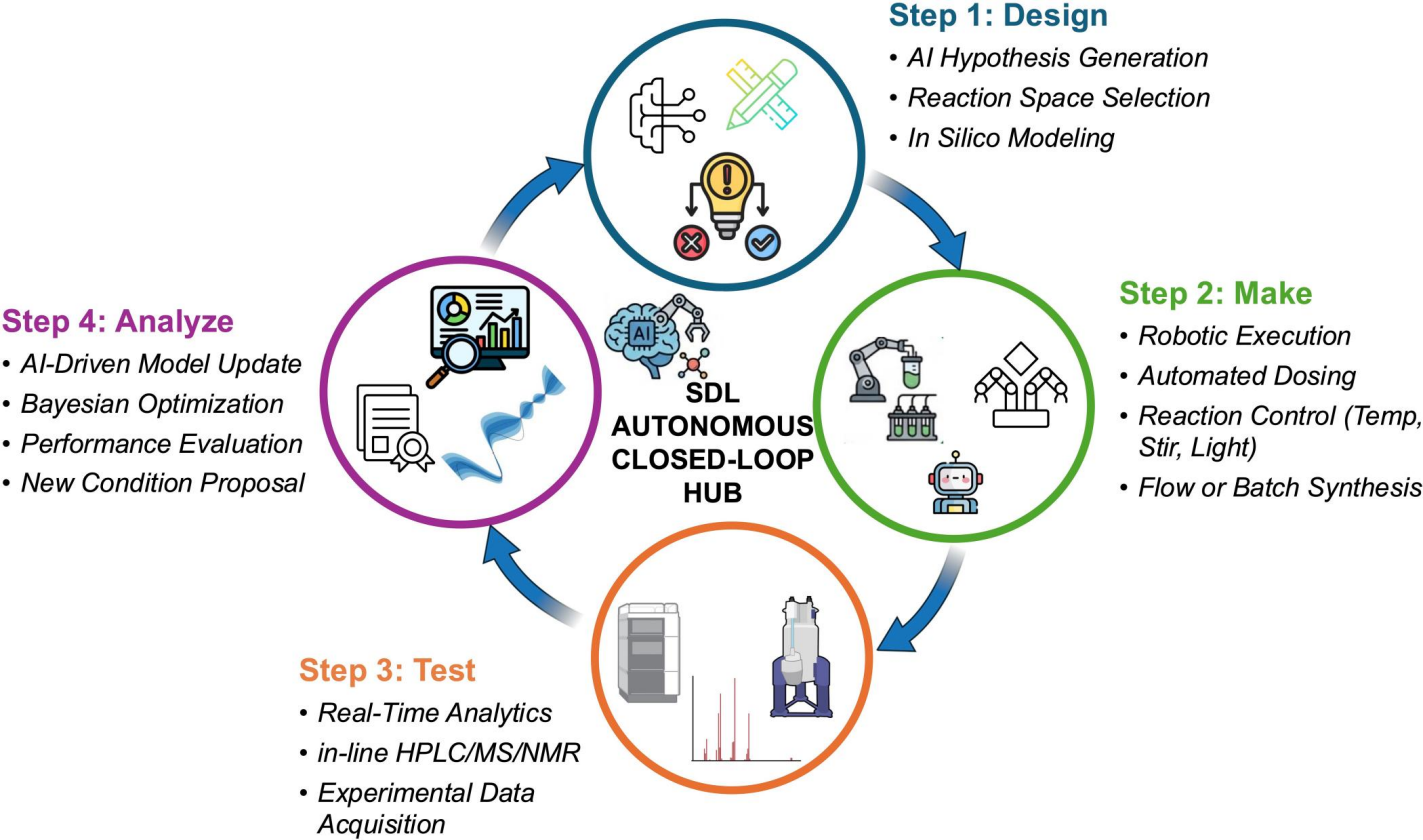
Schematic of a closed‐loop SDL for autonomous chemical synthesis. The workflow follows a fixed sequential order arranged in a circular loop and proceeds clockwise: (Step 1) Design: hypothesis generation and selection of reaction/property space; (Step 2) Make: robotic execution with automated dosing and reaction control; (Step 3) Test: real‐time analytics using in‐line HPLC, MS, NMR (process analytical technology (PAT)); (Step 4) Analyze: AI‐driven model update and proposal of new conditions. Directional arrows indicate both the sequential progression of experimental steps and the feedback loop that connects execution to model updating. [Color figure can be viewed at wileyonlinelibrary.com]

In parallel, ML frameworks guide high‐throughput reaction screening across drug‐like fragments, with active learning and BO algorithms identifying optimal parameter regions efficiently (detailed algorithmic implementations are discussed in Section 2.3.2) [49]. Integration with AlphaFold predictions and Chemistry42 generative design enabled identification of cyclin‐dependent kinase 20 inhibitor scaffolds within 30 days using only seven compounds, supporting cost‐efficient and accelerated decision cycles [50]. These frameworks illustrate the emerging potential of algorithmic reasoning to guide physical experimentation. While currently limited to narrow, well‐characterized reaction classes, these proof‐of‐concept systems aim to eventually support miniaturized, resource‐efficient, and reproducible synthesis more broadly.

Representative robotic infrastructures such as AutoSyn, Chemputer, and the MIT ASKCOS platform exemplify the integration of route planning, flow chemistry, and autonomous execution within a single closed loop [51, 52, 53]. Microfluidic and flow‐based AI systems now perform real‐time reaction optimization through inline analytical monitoring integrated with model‐driven control architectures [54]. Such closed‐loop experimentation reduces trial‐and‐error, enhances reproducibility, and improves laboratory safety. Early platforms by Ley et al. [55] automated hazardous operations, while AutoSyn demonstrated fully automated multistep synthesis of drug‐like small molecules at mg‐to‐g scale within hours and achieved 87% predicted synthesizability across computable FDA‐approved small‐molecule drugs [52]. In parallel, the Chemputer platform validated automated multistep syntheses of pharmaceuticals including rufinamide and sildenafil with yields and purities comparable to or better than manual synthesis [53]. Techno‐economic analyses indicate that closed‐loop execution reduces time, labor, and material consumption by over 30%, supporting green‐by‐design manufacturing.

Ongoing efforts target greener solvent prioritization, automated batch‐to‐flow translation, and intelligent reagent selection. Table 3 summarizes representative robotic and AI‐enabled infrastructures that underpin these integrated DMTA pipelines. By coupling predictive AI, robotic execution, and continuous feedback, these systems enable dynamic design–test cycles with minimal human intervention, with the potential to reduce error and reagent consumption under optimized conditions. Section 2.3.2 describes the algorithmic optimization frameworks that guide these infrastructures toward optimal and sustainable synthesis outcomes.

**Table 3:** Representative AI‐driven and robotic chemistry platforms with applications in medicinal chemistry.

Platform	Key features	Pharmaceutical applications	Notable achievements	Access
MIT ASKCOS Robotic Flow Platform [51]	CASP + modular flow; robotic arm	Small‐molecule APIs; library synthesis	15 drug‐like molecules; yields 32%–95%; stereocontrol	Open‐source software; custom academic hardware
AutoSyn [52]	Fully automated multistep synthesis	Rapid lead compound production	> 87% synthesizability of drug‐like libraries; mg–g scale	Proprietary
Chemputer [53]	Standardized chemical programming	Multistep organic synthesis	Reproducible batch protocols	Open‐source code; proprietary hardware
SDLs [56]	BO‐driven experimentation	Route optimization, yield enhancement	Autonomy supporting > 600 experiments	Varies
Mobile Robotic Chemist [57]	Autonomous BO‐mobile platform	Late‐stage functionalization testing	6× efficiency boost	Proprietary
Synbot [48]	Closed‐loop batch optimization	Optimization of key steps in API synthesis	Improved conversion	Proprietary
RoboRXN [58]	Cloud retrosynthesis + automation	Remote scalable medicinal chemistry	> 29,000 users; > 90% syntactic validity	Proprietary

By integrating AI‐guided DMTA and SDL frameworks, medicinal chemistry now approaches a paradigm where every experiment generates data feedback for algorithmic optimization, a concept further explored in the following sections on reaction optimization algorithms and multi‐objective green synthesis.

#### 2.3.2 BO and Active Learning for Reaction Optimization

Building upon DMTA and SDL execution infrastructures, algorithmic frameworks refine reaction outcomes through iterative, data‐efficient experimentation. This section focuses on BO and active learning strategies that enable closed‐loop reaction optimization with minimal experimental overhead. The Cooper group's mobile robotic chemist exemplifies this approach: over 600 photocatalyst optimization experiments were conducted in 8 days, achieving a six‐fold activity improvement through BO‐guided parameter search [57, 59]. BO enables efficient exploration of high‐dimensional reaction spaces by focusing experimental resources on parameter regions offering maximum predicted improvement or model uncertainty reduction.

Traditional Design of Experiments methods often struggle with multi‐parameter optimization when reaction outcomes depend on non‐linear interactions between temperature, catalyst loading, solvent composition, and reaction time. Data‐driven frameworks address these challenges by constructing probabilistic surrogate models (typically Gaussian Processes [GPs]) that predict reaction outcomes and guide experimental design toward regions of high uncertainty or high predicted performance. These approaches have accelerated reactions such as C–N cross‐coupling [60] and low‐temperature Suzuki–Miyaura couplings [61].

Flow photochemistry platforms integrated with AI‐guided BO improve photon efficiency and convergence, reflecting sustainability potential. Structured reaction datasets such as the Open Reaction Database (ORD) [62] provide the data‐efficiency backbone supporting reproducible model development. Industrial platforms such as IBM RoboRXN [58] and AI4Green [63] exemplify the transition toward distributed, solvent‐efficient, and green‐by‐design synthesis optimization.

Within this framework, BO serves as a cornerstone for optimizing black‐box objectives, balancing yield, selectivity, and environmental impact [64]. GP surrogates quantify uncertainty, while acquisition functions enable MOO of reaction performance and sustainability [65]. Bayesian neural networks and multi‐fidelity extensions expand these methods to diverse reaction families, improving generalization across scales.

AI algorithms iteratively propose diverse candidate conditions, which are experimentally tested and refined [66, 67]. Platforms like Experimental Design via BO [8, 68] enable multi‐objective BO, balancing yield, selectivity, cost, and time. Closed‐loop high‐throughput experimentation (HTE) systems employing algorithms such as Phoenics [69] and Gryffin [70] have demonstrated robust performance across diverse reaction classes, extending seamlessly to medicinal chemistry screening arrays [71].

Table 4 summarizes key AI and MOO algorithms integrated within HTE and SDL infrastructures, spanning single‐reaction optimization (BO, active learning) and multi‐objective retrosynthetic planning (GA, MORL). This unified framework illustrates how algorithmic strategies at different scales converge to support sustainable, data‐efficient medicinal chemistry workflows. HITL approaches, discussed in detail in Section 2.3.4, combine algorithmic efficiency with chemists' intuition, ensuring interpretability and reliability.

**Table 4:** Key AI and multi‐objective optimization algorithms in reaction and retrosynthetic design.

Algorithm/model	Core technique	Primary application	Key features	Typical use cases	Integration with HTE/SDL/sustainability
Bayesian optimization (BO)	Probabilistic surrogate (Gaussian Process or TPE)	Reaction condition tuning	High data‐efficiency; uncertainty‐driven exploration; adaptable to multi‐objective tasks	C–N, Suzuki–Miyaura couplings, flow photochemistry	Core for HTE/SDL; supports energy and solvent optimization
Active learning (AL)	Iterative model‐driven experiment selection	Reaction space exploration	Minimizes failed experiments; efficient in sparse data regimes	Catalyst and solvent screening	Excellent HTE/SDL synergy; sustainability alignment
Experimental design via BO (EDBO+)	Multi‐objective Bayesian optimization	Yield–cost–time trade‐offs	Pareto‐efficient optimization; interpretable performance metrics	Photocatalyst optimization; screening arrays	Streamlined HTE integration; energy efficiency gains
Genetic algorithms (e.g., NSGA‐II)	Evolutionary selection, crossover, mutation	Multi‐objective route planning	Pareto‐based exploration; highly parallelizable	Sonogashira, Claisen–Schmidt couplings	High scalability; robust for green metrics trade‐offs
Multi‐objective reinforcement learning (MORL)	Policy‐based adaptive learning via rewards	Dynamic retrosynthetic planning	Learns under changing objectives; scalable on graph spaces	Scaffold and route optimization	Integrates real‐time sustainability penalties; SDL‐ready
Transfer/few‐shot learning	Pretrained deep networks; GNN/Transformer embeddings	Low‐data reaction families	Fast convergence; plausible novel condition prediction	Organocatalysis, electro/photo‐chemistry	Ideal for early‐stage discovery; sustainability pending
HITL optimization	BO/AL + expert feedback	All reaction variables	50%–70% faster convergence vs. grid search; interpretable	Lead optimization, late‐stage screening	Chemist‐in‐loop validation; resource‐efficient
Large‐scale recommenders	Transformer/ensemble models	Condition suggestion across corpora	Rapid search‐space reduction (~30%)	Broad reaction coverage	Useful pre‐filter for HTE/SDL; flow‐compatible
Ensemble/Chimera Frameworks	Multi‐model fusion	Robust MOO under sparse data	Captures under‐represented chemistries	Late‐stage route design	Enhances reliability, sustainability benchmarking

#### 2.3.3 MOO for Green and Sustainable Retrosynthesis

While Section 2.3.2 focused on single‐reaction optimization, medicinal chemistry synthesis planning requires simultaneous optimization of multiple, often competing objectives: yield, cost, step count, environmental impact, and scalability. This section describes MOO frameworks that enable explicit trade‐off analysis and Pareto‐optimal route selection.

MOO frameworks identify synthetic routes that optimize trade‐offs across competing goals through algorithms such as genetic algorithms (GA), BO‐based approaches, and MORL [72]. Advanced strategies, including Thompson Sampling, Efficient Multi‐Objective Optimization and Phoenics, have improved sample efficiency and convergence in benchmark reactions such as Sonogashira couplings and Claisen–Schmidt condensations [73, 74, 75].

The Pareto front is central to MOO: it represents the set of solutions where improvement in one objective necessitates degradation in another. In retrosynthetic planning, each point on the Pareto front represents a chemically feasible route with distinct trade‐offs between cost, environmental impact, step count, and predicted yield. Metrics such as hypervolume and route diversity quantify overall performance, while interactive dashboards support dynamic weighting of trade‐offs, particularly for green chemistry objectives. Figure 3 illustrates how Pareto fronts visualize trade‐offs between cost and environmental impact at fixed yield, helping chemists identify synthetic routes that align with both economic and green metrics priorities.

**Figure 3:**
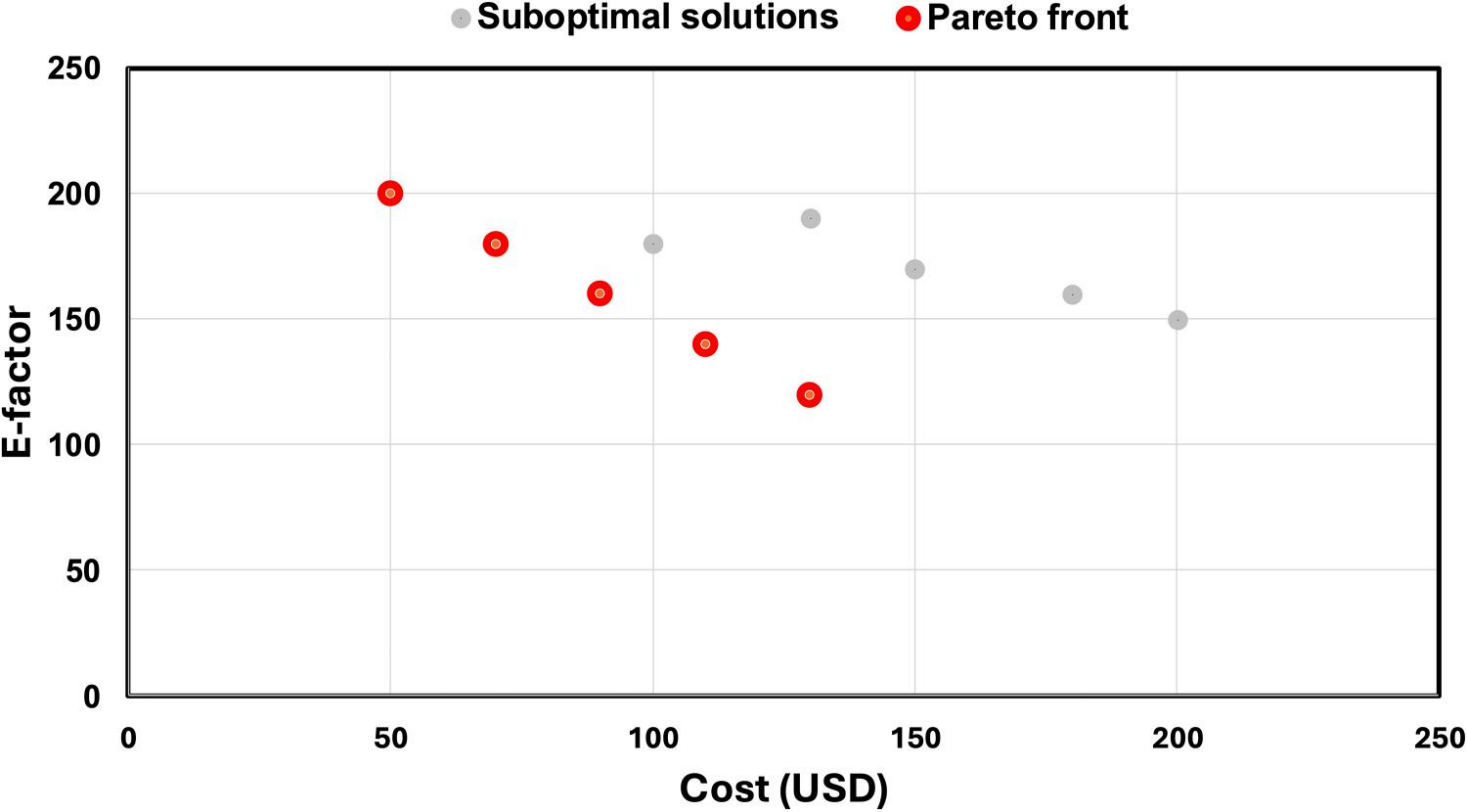
Pareto front in multi‐objective retrosynthetic planning. Each point represents a synthetic route plotted by its cost and environmental impact, assuming constant yield. Axes are labeled as follows: x = USD per mmol (monetary cost) and y = E‐factor (kg waste per kg product). Red points denote Pareto‐optimal routes, while gray points represent suboptimal alternatives. This visual framework helps chemists navigate trade‐offs such as cost versus sustainability when selecting synthesis routes, thereby supporting informed decision‐making in AI‐driven retrosynthesis. [Color figure can be viewed at wileyonlinelibrary.com]

Orforglipron (LY3502970), a glucagon‐like peptide 1 receptor agonist, exemplifies the practical impact of AI‐guided MOO in retrosynthetic planning. Figure 4 illustrates the comparison between the original synthetic route and the AI‐optimized alternative, providing a direct and visually guided comparison of step count reduction and pathway simplification. Using the ChemAIRS platform, an AI‐guided analysis reduced the patented 28‐step synthetic route to 16 steps, revealing opportunities for cost reduction, faster throughput, and improved environmental efficiency. Key intermediates (dihydro‐imidazol‐2‐one and indole‐2‐carboxylic acid) were shortened from 15 → 8 and 11 → 7 steps, respectively. The model predicts reaction outcomes, side products, and optimal conditions, supporting the identification of more efficient synthetic pathways. Coupled with robotic HTE, this approach enables rapid validation while minimizing reagent use and waste generation (Figure 4).

**Figure 4:**
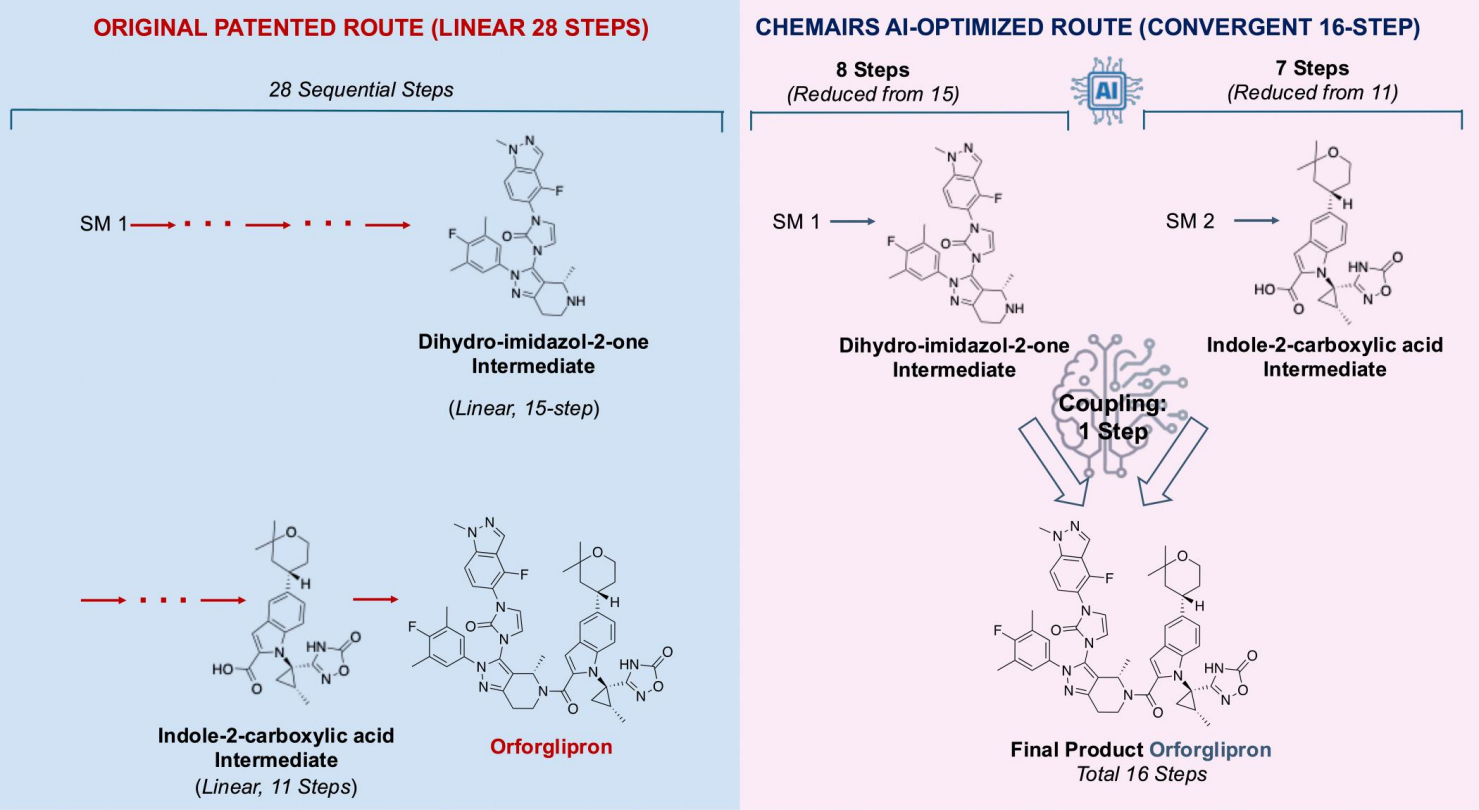
AI‐optimized synthetic route for Orforglipron (LY3502970). Comparison between the original 28‐step patented route and the 16‐step AI‐optimized pathway generated by ChemAIRS, presented in a side‐by‐side layout to enable direct visual comparison, highlighting key intermediates and step reductions (15 → 8 and 11 → 7), along with resource optimization and automation‐readiness indicators. Key step reductions and pathway simplifications are explicitly annotated to facilitate intuitive interpretation of efficiency gains. SM, starting material. Data are derived from Chemical. AI's publicly reported example (https://www.chemical.ai/blog/retrosynthetic-strategy-for-orforglipron-ly3502970-using-chemairs-developing-a-more-streamlined-and-efficient-synthetic-route-suggestion). [Color figure can be viewed at wileyonlinelibrary.com]

In retrosynthesis, MOO enables explicit optimization of drug‐relevant criteria, including cost, yield, step count, environmental impact, and scalability [76, 77]. Interactive visual analytics support decision‐making, enabling chemists to explore Pareto‐optimal pathways and tailor trade‐offs to pharmaceutical constraints such as timeline, impurity control, and supply chain resilience. Techno‐economic analyses of continuous manufacturing processes confirm material and time savings relative to batch operations [78].

Among MOO algorithms, GAs, particularly Non‐dominated Sorting Genetic Algorithm II (NSGA‐II) [79], along with BO‐based approaches and MORL [80], are widely applied. These methods simulate evolutionary processes (selection, crossover, mutation) to balance synthetic feasibility, cost, and sustainability in route selection. Open‐source frameworks such as DEAP and Platypus provide accessible implementations [81]. Detailed algorithm characteristics, including data efficiency, parallelizability, and integration potential, are summarized in Table 4.

Recent uncertainty‐aware planners penalize pathways predicted to be synthetically infeasible, improving reliability under low‐data conditions [82]. MORL introduces adaptive, reward‐based learning that is especially effective for complex retrosynthetic graphs [80]. Agents iteratively interact with a simulated chemical environment, learning policies that favor high‐yield, short‐step, and low‐waste pathways while penalizing toxic reagents or harsh conditions [83, 84].

The synergy of NSGA‐II and MORL enables dynamic route optimization under evolving project priorities, minimizing late‐stage inefficiencies. In pharmaceutical contexts, these methods have optimized catalytic transformations critical to active pharmaceutical ingredient (API) synthesis, such as Suzuki–Miyaura and amide couplings, where maximizing conversion, stereoselectivity, and impurity control is essential.

Ensemble frameworks, including Chimera [85], enhance robustness by integrating models with complementary inductive biases, capturing underrepresented transformations common in medicinal chemistry scaffolds. Transformer‐based architectures combined with many‐objective optimization extend benefits into late‐stage design, generating molecules satisfying binding affinity, predicted ADMET, and synthetic feasibility [86]. Innovations such as Pareto Set Learning improve computational efficiency by decomposing complex MOO problems into solvable sub‐tasks [87].

Together, these advances demonstrate that yield‐only optimization is no longer sufficient. Route quality, scalability, and sustainability are now integral determinants of regulatory and commercial success. Sustainability objectives, including energy efficiency, solvent greenness, and catalyst recyclability, are embedded directly in MOO frameworks, aligning with SDL architectures for real‐time feedback and waste reduction [88]. Recent digital platforms such as AI4Green [63] and GreenRoute [89] employ MOO algorithms that balance yield, selectivity, and environmental impact, often incorporating one‐pot, telescoped, or continuous‐flow strategies to enhance overall resource efficiency. Benchmarking studies indicate that these systems can reduce the effective design space by approximately 25%–40% and accelerate identification of low‐waste pathways, although validation remains limited to well‐characterized reactions [90].

#### 2.3.4 Critical Comparative Analysis of AI Methodologies

AI methodologies for chemical synthesis can be broadly categorized into retrosynthesis models, optimization algorithms, and emerging LLM‐based frameworks. While the rapid proliferation of these approaches has enabled significant advances, a comparative analysis reveals clear operational trade‐offs that determine their suitability across different stages of medicinal chemistry workflows.

Template‐based retrosynthesis methods remain the most reliable in industrial and late‐stage settings due to their high chemical validity, interpretability, and alignment with known reaction rules. However, their dependence on predefined transformations limits their ability to explore novel chemical space. As a result, these approaches are particularly suited for late‐stage optimization and process chemistry, but are less effective for early‐stage scaffold exploration and innovation. In contrast, template‐free models, typically based on transformer or GNN architectures, enable greater structural diversity and innovation by learning directly from data, but often suffer from reduced reliability, including chemically implausible predictions and sensitivity to training data bias [39, 91]. These limitations become especially pronounced in out‐of‐distribution scenarios, such as stereochemically complex molecules, natural products, or macrocycles.

Hybrid or semi‐template approaches attempt to balance these trade‐offs by combining data‐driven prediction with mechanistic constraints. These methods improve chemical validity relative to template‐free models while retaining greater flexibility than purely rule‐based systems, although they introduce additional computational complexity and may struggle with multi‐center transformations.

Recent LLM‐based systems extend retrosynthesis beyond prediction toward reasoning and workflow orchestration. While they enable integration of literature knowledge, reaction conditions, and multi‐step planning, their generalized architecture limits precision in reaction‐level predictions and introduces risks of hallucination and insufficient chemical grounding. As a result, they are currently best positioned as decision‐support tools rather than standalone synthesis planners [39, 40]. Their performance also remains dependent on external validation tools and structured pipelines to ensure chemical plausibility and experimental feasibility.

For reaction optimization, BO represents the most data‐efficient approach, particularly in low‐data experimental settings, enabling targeted exploration of high‐dimensional parameter spaces [8]. Active learning strategies further enhance this process by prioritizing the most informative experiments, improving convergence in data‐sparse regimes. In contrast, evolutionary algorithms and reinforcement learning offer advantages in multi‐objective and adaptive optimization but typically require higher computational or experimental cost and may exhibit reduced robustness in practical laboratory settings.

A key distinction also exists between LLM‐based systems and classical ML models such as GNNs and transformer‐based predictors. While classical models typically achieve higher predictive accuracy in well‐defined tasks such as reaction prediction and yield estimation, LLMs offer broader reasoning capabilities and cross‐task integration, albeit with reduced precision and higher uncertainty. This distinction reflects a fundamental trade‐off between task‐specific accuracy and general‐purpose reasoning: while classical models excel in well‐defined predictive tasks, LLM‐based systems provide flexible, context‐aware support across multiple stages of synthesis planning but require additional validation to ensure chemical correctness, particularly in experimentally constrained settings. Overall, no single methodology consistently outperforms others across all synthesis tasks, and each approach occupies a distinct operational niche defined by trade‐offs between interpretability, generalizability, data efficiency, and experimental reliability. In practice, robust workflows increasingly rely on integrated strategies that combine complementary methodologies with human expertise. From an expert perspective, a key limitation remains the fragmentation of current AI methodologies across prediction, optimization, and execution tasks, which are often treated independently rather than as components of unified synthesis workflows. Addressing this fragmentation represents a critical step toward translating AI‐driven synthesis into routine medicinal chemistry practice.

#### 2.3.5 Human–AI Collaborative Optimization

The convergence of algorithmic optimization with human expertise through HITL frameworks represents a critical enabler for translating AI predictions into reliable experimental protocols, particularly in the context of the methodological trade‐offs outlined in Section 2.3.4. This section describes how substrate‐aware feedback, transfer learning, and interactive decision‐support tools enhance the robustness and interpretability of AI‐driven synthesis optimization.

Substrate‐aware feedback mechanisms within HITL loops enable early detection and correction of mispredicted conditions, thereby enhancing robustness in SAR interpretation and revealing critical developability constraints such as solubility limits, redox instability, or residual metal contamination [91, 92].

HITL approaches combine the sample efficiency of BO and active learning (Section 2.3.2) with chemists' mechanistic intuition, domain knowledge, and risk assessment capabilities. Interactive platforms allow chemists to modify algorithmic suggestions, flag safety concerns, or incorporate proprietary knowledge that is not captured in public datasets. This bidirectional feedback loop improves model calibration, reduces experimental failures, and maintains interpretability throughout the optimization cycle.

Industrial case studies highlight both opportunities and limitations of HITL integration. At AstraZeneca, the hybrid AI model SoBo, combining cheminformatics, density functional theory (DFT), and ML, has guided site‐selective arene borylation, a crucial late‐stage functionalization [93]. By predicting regioselectivity more accurately than human experts, SoBo has minimized protecting‐group manipulations and reagent excess, thereby reducing waste and improving energy efficiency. However, AI‐based route selection may sometimes prioritize theoretically low‐waste yet experimentally fragile conditions, emphasizing the need for HITL validation and iterative refinement to ensure reproducibility and robustness. For example, data‐driven optimization frameworks have been shown to favor reaction conditions that maximize predicted yield or minimize waste, but may rely on catalysts, solvents, or parameter combinations that are highly sensitive to experimental variability, catalyst instability, or scale‐up constraints [91]. These limitations illustrate the gap between computational optimality and practical robustness, reinforcing the importance of expert‐driven validation.

In summary, HITL frameworks ensure that AI‐driven reaction optimization remains grounded in experimental reality, chemical safety, and regulatory requirements, bridging the gap between algorithmic predictions and scalable manufacturing processes.

#### 2.3.6 Industrial Case Studies in AI‐Guided Green Chemistry

The industrial deployment of AI‐driven sustainable synthesis strategies is exemplified by case studies demonstrating measurable improvements in environmental metrics, cost‐efficiency, and process robustness. Building on the algorithmic frameworks discussed in Sections 2.3.2–2.3.4, this section illustrates how AI‐driven sustainability translates into tangible process improvements at industrial scale.

AstraZeneca's AI‐guided late‐stage functionalization and photocatalytic platforms eliminated 3–5 synthetic steps per molecule while replacing palladium with cobalt or nickel catalysts, achieving 40%–60% waste reduction and improving E‐factors from 50–100 to 15–30. Miniaturized, automated reaction systems reduced solvent consumption by approximately 30%–50% and enabled more than 1000‐fold decreases in waste per exploratory reaction (https://www.astrazeneca.com/what-science-can-do/topics/sustainability/sustainable-drug-discovery-using-green-chemistry.html).

These achievements demonstrate that embedding sustainability metrics (e.g., step count, atom economy, solvent toxicity, life‐cycle impact) into AI reward functions can yield quantifiable environmental and cost benefits. However, large‐scale deployment under Good Manufacturing Practice (GMP) conditions still demands stringent validation to ensure reproducibility, robustness, and regulatory compliance. These implementations illustrate how explainability and regulatory readiness, discussed in Section 4, become critical to scaling AI‐driven green chemistry under industrial constraints. Together, they signal a shift toward sustainability‐by‐design drug discovery, closing the loop between algorithmic intelligence and responsible manufacturing. This convergence of robotic infrastructures (Section 2.3.1), algorithmic optimization (Sections 2.3.2), human–AI collaboration (Section 2.3.4), and industrial validation collectively demonstrates that modern medicinal chemistry synthesis is transitioning toward fully integrated, feedback‐driven, and sustainability‐aware workflows. Section 4 extends these principles to end‐to‐end drug discovery case studies, quantifying the impact of AI‐enabled synthesis across target identification, lead optimization, and clinical development.

## 3 Autonomous Robotic Platforms and Automated Execution in Synthesis

The translation of AI‐driven retrosynthetic planning into tangible pharmaceutical assets represents a critical bottleneck in modern medicinal chemistry. Within the hierarchical framework introduced in this review, this section focuses on the physical execution layer, where digitally generated synthetic plans are translated into experimentally validated outcomes through robotic and automated systems. This section evaluates how closed‐loop robotic systems and automated experimental platforms are being developed to address this barrier, with increasing evidence of their practical potential. Recent case studies illustrate the potential and current limitations of integrating AI with automated synthesis hardware, with a particular emphasis on robotic platforms, closed‐loop experimentation, and autonomous execution systems. These studies report measurable improvements in speed, material efficiency, and sustainability, while also highlighting persistent challenges in hardware scalability and chemical diversity.

Table 5 summarizes representative examples and their quantitative synthesis metrics.

**Table 5:** Representative case studies of AI‐enabled synthesis: Key metrics and lessons learned.

Case	Organization/system	Key quantitative impact	Outcome	Primary limitations	Lessons learned
INS018_055 (TNIK inhibitor)	Insilico Medicine	Timeline: 30 mo vs. 4.5–6 yrs (↓60%–70%) • < 80 compounds vs. 1–5 k (↓ 92%–98%) • Cost < $10 M vs. $40–80 M	Phase IIa efficacy in 71 patients (IPF)	Relies on validated target; synthetic accessibility prioritized over novelty	Integrate target–design–synthesis workflows early; embed ADME and manufacturability filters; human oversight remains critical
DSP‐1181/EXS21546	Exscientia	Discovery ≤ 12 mo (↓70%–75%) • 150–250 compounds (↓88%–93%)	DSP‐1181 discontinued (Phase I); EXS21546 ongoing	Limited structural novelty; biology‐driven attrition	Speed ≠ innovation; ensure chemical diversity; use automation for closed DMTL loops
Autonomous mobile robots	Univ. of Liverpool	10–20× faster decision cycles; 1–5 mL miniaturized reactions	Proof‐of‐concept – reproducible autonomous chemistry	Screening‐scale only; no GMP compliance	Decision logic > hardware; modular robotics feasible for early exploration
AI‐Designed molecules (general failures)	Multiple (BenevolentAI, Recursion)	—	Phase II attrition; poor efficacy/PK	Insufficient negative data; lab‐to‐manufacturing gap	Curate failed data; hybrid human–AI validation; engage process chemists early
Green chemistry platforms	AstraZeneca	Route shortening 3–5 steps • E‐factor 50–100 → 15–30 (↓60%–70%) • Solvent use ↓ 30%–50%	Portfolio‐scale sustainability improvements	Regulatory scaling under GMP	Embed green metrics into AI optimization; involve process chemists from the discovery stage

### 3.1 AI‐Accelerated Medicinal Chemistry Synthesis Workflows in the Design‐Make‐Test‐Learn (DMTL) Cycle

In recent successful discovery campaigns, the most significant efficiency gains have emerged from optimizing the “Make” phase within the DMTL cycle. In this context, AI‐driven synthesis planning and execution play an increasingly important role by prioritizing synthetically accessible compounds and enabling their rapid experimental realization through automated workflows. For example, in the development of INS018_055, the integration of generative design with strict synthetic feasibility filters enabled the team to synthesize fewer than 80 compounds to achieve a viable Phase II candidate. This represents a 92%–98% reduction compared to traditional campaigns, although such improvements are case‐specific and not yet generalizable across all medicinal chemistry programs, as supported by recent clinical and methodological reports [94, 95]. This drastic reduction in laboratory experimentation conserved reagents and solvents. It also translated into substantial synthesis‐related cost savings and a highly compressed 30‐month timeline. Importantly, these results were supported by experimental validation of AI‐prioritized compounds, demonstrating that computationally selected candidates could be successfully synthesized and tested within accelerated timelines. These results highlight that the primary impact of AI in these workflows arises from improved synthesis prioritization, reduced synthetic burden, and tighter integration between computational design and experimental execution.

Similarly, Exscientia's development of DSP‐1181 relied on a closed‐loop DMTL platform that paired multi‐parameter algorithmic scoring with robotic synthesis capabilities. By explicitly constraining the AI to prioritize synthetically accessible and automatable routes, the workflow required the physical synthesis of only 150–250 compounds, advancing the candidate to Phase I in approximately 12 months. This workflow therefore represents a fully integrated example of AI‐assisted synthesis in which retrosynthetic planning, compound prioritization, and experimental validation were tightly coupled within a closed‐loop system. While DSP‐1181 was ultimately discontinued, the campaign successfully validated the premise that predictive retrosynthesis and automated execution can effectively bypass traditional synthesis bottlenecks. These experiences underscore that AI's primary value in synthetic chemistry lies in preventing the physical execution of redundant or low‐probability routes, thereby maximizing the efficiency of automated laboratory infrastructure.

Collectively, these examples demonstrate that AI contributes to drug discovery not only through molecular design but, critically, through experimentally validated improvements in medicinal chemistry synthesis workflows, including reduced compound synthesis, accelerated iteration cycles, and more efficient resource utilization.

### 3.2 Autonomous Robotic Platforms and Experimental Acceleration

Beyond algorithmic design, robotic systems are increasingly closing the experimental loop. To reflect the diversity of current approaches, this section highlights complementary robotic architectures, including mobile platforms, modular flow systems, and closed‐loop self‐optimizing reactors. The University of Liverpool's mobile robotic chemist demonstrated autonomous decision‐making through AI control, Chemspeed synthesis, and on‐board liquid chromatography–mass spectrometry/NMR analytics [96]. These robots executed reaction optimization and scale‐up decisions 10–20 times faster than human chemists while maintaining reproducibility across urea and copper‐catalyzed azide–alkyne cycloaddition reactions. Miniaturized reaction volumes (1–5 mL) and intelligent pre‐screening strategies minimized waste and solvent consumption. Although scalability and GMP compliance remain open challenges, this proof‐of‐concept highlights that decision logic (the AI‐driven interpretation of analytical data into synthetic choices) provides more value than hardware automation alone. Such modular and adaptable systems offer practical entry points for smaller organizations exploring robotic synthesis without a complete infrastructure overhaul.

To address the hardware limitations of traditional linear or iterative automated syntheses, Chatterjee et al. [97] developed a versatile approach utilizing continuous flow modules arranged radially around a central switching station. This fully automated radial instrument allows for sequential, non‐simultaneous reactions to be combined for multistep processes without requiring any manual reconfiguration between different synthetic steps. The capabilities of this central hub approach were extensively validated through the multistep synthesis of the anticonvulsant drug rufinamide, the preparation of two derivative libraries using varying reaction pathways, and the execution of metallaphotoredox carbon‐nitrogen cross‐couplings in a photochemical module [97].

Furthermore, recent advancements have targeted the specific challenges of optimizing, intensifying, and scaling photochemical processes in flow. Slattery et al. [98] introduced “RoboChem,” a benchtop robotic platform that integrates a liquid handler, syringe pumps, a tunable continuous‐flow photoreactor, and an in‐line benchtop NMR spectrometer. By employing a closed‐loop BO strategy, RoboChem systematically explores parametric spaces to autonomously identify conditions that maximize reaction yield, throughput, or a combination of both. The platform's high precision and reliability were demonstrated across 19 different molecules covering diverse facets of photocatalysis, consistently identifying optimal reaction conditions that matched or exceeded human‐optimized manual approaches while generating highly structured datasets of both positive and negative results [98].

Recent end‐to‐end autonomous platforms integrating LLMs with robotic execution further illustrate this trend toward fully automated synthesis development pipelines [99].

Collectively, these platforms illustrate that the integration of intelligent decision algorithms with adaptive hardware provides significant practical value by bridging the gap between digital retrosynthesis planning and physical, scalable execution, reflecting the transition of autonomous synthesis from proof‐of‐concept demonstrations to increasingly robust and generalizable experimental frameworks.

### 3.3 Failures, Data Gaps, and Lessons Learned for Medicinal Chemists

Not all AI‐designed molecules have succeeded. Programs from BenevolentAI, Recursion, and Exscientia have faced attrition at Phase II due to efficacy or pharmacokinetic liabilities unpredicted by AI models. Common causes include insufficient negative training data, limited representation of failed experiments, and over‐optimization for synthetic accessibility at the expense of novelty. Publication bias toward successful outcomes further skews datasets, underrepresenting toxicity, instability, and scale‐up challenges [100].

Despite notable advances, retrosynthesis algorithms still exhibit approximately 70%–75% single‐step accuracy, meaning roughly one in four AI‐proposed routes fails experimentally. Typical errors include reaction‐condition generalization, stereochemical mispredictions, and unrealistic precursor assumptions. Hybrid human–AI approaches, where chemists validate algorithmic suggestions, significantly improve success rates and maintain scalability [11, 101]. Ultimately, AI accelerates exploration but cannot substitute for mechanistic understanding or process‐chemistry expertise, particularly in complex or data‐sparse chemical spaces. Early involvement of manufacturing scientists remains essential to ensure scalability and regulatory compliance. From a critical perspective, current AI‐driven synthesis platforms remain highly effective in well‐defined chemical spaces but show limited robustness when extrapolated to novel scaffolds, complex stereochemical systems, or underrepresented reaction classes. This discrepancy highlights a persistent gap between algorithmic performance metrics and real‐world medicinal chemistry requirements, where failure modes, edge‐case behavior, and experimental uncertainty play a central role. Addressing this gap requires improved integration between computational prediction, experimental validation, and process‐level considerations. In our view, this also necessitates a cultural shift toward systematic reporting of failed experiments and negative data, which remain severely underrepresented in current training datasets and continue to limit model robustness.

Across these diverse case studies, several consistent lessons emerge for medicinal chemists adopting AI‐enabled synthesis:

1. Data‐laboratory integration is essential; curated reaction data, including negative results, must feed back into model refinement.
2. Human expertise remains central: interdisciplinary teams of synthetic, computational, and process chemists must jointly assess AI outputs before committing laboratory resources.
3. Regulatory readiness and transparency are crucial; explainable models, impurity tracking, and decision logs are prerequisites for GMP translation.
4. AI delivers the highest value in large or data‐rich chemical spaces, while genuinely novel or data‐sparse targets still depend on chemists' intuition.
5. Embedding green chemistry metrics directly into design objectives ensures innovation aligns with environmental and corporate sustainability goals.

AI and robotics are beginning to influence medicinal chemistry practice; in specific case studies, discovery timelines have been reduced by 60%–80%, synthesis counts by over 90%, and early‐stage costs by approximately 75%–85%. However, these improvements remain case‐specific and are not yet broadly generalizable across diverse chemical targets and discovery programs. Yet, these quantitative gains highlight enduring qualitative challenges: algorithmic bias, limited scaffold diversity, and the irreplaceable role of human judgment. The future of medicinal chemistry will depend on coupling automated design and synthesis with rigorous data stewardship, process insight, and sustainability foresight, enabling chemists to explore chemical space more intelligently, responsibly, and at unprecedented speed.

## 4 Interpretable and Regulatory‐Ready Retrosynthesis

Continued progress in AI‐driven synthesis requires transparent benchmarking, sustainability‐aware objectives, and deeper integration with SDL infrastructures, supporting the emergence of sustainability‐by‐design medicinal chemistry ecosystems. Explainable AI (XAI) has become indispensable in AI‐guided retrosynthesis, ensuring that synthetic predictions are trustworthy, chemically interpretable, and experimentally auditable [102]. Clarity and interpretability are critical in medicinal chemistry, where synthetic choices affect project timelines, resource allocation, intellectual property strategy, safety, and manufacturing feasibility during preclinical and clinical development. Transparent architectures capable of justifying each disconnection strategy, through rule‐based systems, decision trees, or hybrid AI frameworks, are preferred over purely confidence‐based black‐box models, particularly due to the high cost of experimental failures during late‐stage scale‐up [103].

### 4.1 The Role of XAI in Medicinal Chemistry

Beyond optimization, interpretability and validation metrics ensure that AI‐guided retrosynthesis is transparent, auditable, and regulatory‐ready. However, rapid algorithmic progress has not always been matched by methodological rigor. Claims of generality require systematic benchmarking, ablation studies, and transparent dataset reporting to ensure true generalization rather than overfitting. Chemically meaningful validation metrics (yield, robustness, scalability) should complement conventional accuracy scores to reflect laboratory performance. Interpretability enhances synthetic risk assessment, identifies dataset biases, and supports regulatory traceability [104]. Because attention weights or raw confidence scores can be misleading when reported without mechanistic context [105, 106], best practice combines multiple explainability modalities that highlight plausible bond disconnections, leaving‐group behavior, and reagent compatibilities, while explicitly reporting uncertainty sources and data limitations.

In medicinal chemistry, this transparency bridges algorithmic predictions with chemical intuition, enabling experts to evaluate whether proposed routes are safe, scalable, and compliant with GMP. Transparent reasoning is also essential for knowledge transfer and reproducibility, particularly when models are used to support regulatory submissions.

### 4.2 Interpretable Retrosynthesis Models

Recent advances in AI‐assisted retrosynthesis can be broadly categorized into template‐based, semi‐template‐based, and template‐free approaches, each offering distinct advantages and limitations. Template‐based methods rely on predefined reaction rules derived from known transformations, providing high interpretability but limited generalization to novel chemistries. Template‐free approaches, often based on neural sequence‐to‐sequence or graph‐based models, offer greater flexibility and broader chemical coverage but may lack interpretability and mechanistic transparency. Hybrid and semi‐template‐based strategies attempt to balance these trade‐offs by integrating data‐driven learning with chemically meaningful constraints. Comprehensive recent reviews have highlighted how these methodological classes define the current landscape of retrosynthesis research and the key challenges associated with their practical deployment in medicinal chemistry [107].

Within this landscape, interpretable retrosynthesis frameworks aim to bridge predictive performance with mechanistic transparency, addressing key limitations of purely data‐driven approaches.

The methodological framework developed by Wang et al. [108, 109] illustrates a progressive evolution of mechanism‐informed retrosynthesis models. The group initially introduced MechRetro [109], a graph‐based architecture that integrates mechanistic reasoning with uncertainty estimation, achieving approximately 65% recovery of chemically plausible routes on curated datasets, although its validation remains limited for industrial‐scale applications. Building upon these foundational principles, the same group subsequently developed RetroExplainer [108], which represents a direct methodological extension of the MechRetro framework rather than an independent approach, by formulating retrosynthesis as a molecular assembly task using a multi‐sense, multi‐scale Graph Transformer combined with contrastive learning. This approach achieves an 86.9% single‐step literature match across 12 benchmarks, representing a significant methodological advancement. However, despite this improvement, its performance remains influenced by literature bias, which limits generalization to unexplored chemical spaces.

BioNavi, a hybrid neural and template‐based system, reached 75% success in biosynthetic pathway reproduction and supports interactive pathway exploration, although expert intervention remains necessary for resolving ambiguous steps [110]. Alternative approaches such as Graph2Edits [19] adopt graph‐editing strategies with different trade‐offs between interpretability and generalization. Graph2Edits, a semi‐template graph‐editing model, enables sequential fragment modifications with interpretable edits, though its mechanistic insight is limited by graph‐edit definition. Table 6 summarizes leading interpretable retrosynthesis systems, including related models that represent successive developments within the same methodological lineage, detailing architecture, interpretability approach, strengths, limitations, and benchmark performance.

**Table 6:** Comparison between interpretable retrosynthesis models.

Model/framework	Architecture type	Interpretability method	Strengths	Limitations	Verified performance (top‐k accuracy)^a^
Molecular transformer [106]	Transformer (SMILES‐to‐SMILES)	Attention visualization, calibrated confidence estimates	Template‐free; strong generalization and uncertainty estimation	Attention not mechanistic; struggles with rare chemistries; often fails on condition‐sensitive or multicomponent reactions	Forward reaction prediction: 90.4% top‐1 accuracy on the USPTO benchmark reported by the authors; uncertainty calibration AUC = 0.89. Not directly comparable with retrosynthesis‐specific USPTO‐50K top‐k benchmarks.
RetroXpert [111]	Two‐stage: synthon decomposition + template ranking	Explicit synthon‐based rationale	High single‐step accuracy; interpretable	Restricted by template set; struggles with multistep synthesis generalization	USPTO‐50K exact‐match accuracy: 70.4% top‐1 with reaction class provided; 65.5%–65.6% top‐1 without reaction class.
LocalRetro [112]	Template‐based graph transformer	Literature‐derived template rationale	High route validity; interpretable	Poor novel reaction flexibility; fails to generalize to chemistries absent from the template set	USPTO‐50K: 89.5% top‐1 and 99.2% top‐5 round‐trip accuracy; reported as round‐trip validation rather than standard top‐k exact‐match retrosynthesis accuracy.
SCROP [19]	Template‐free + post‐generation corrector	Syntax and valence self‐correction	Reduces invalid SMILES; improves accuracy	May still suggest impractical steps	USPTO‐50K: 59.0% top‐1 accuracy under the benchmark setting reported by the authors; invalid SMILES reduced from 12.1% to 0.7%. Top‐k values are protocol‐dependent and should not be directly compared without specifying reaction‐class input.
BioNavi [110]	Hybrid DL + templates	Template + multitask outputs + UI	Effective biosynthetic route recovery via interactive UI	Relies on curated templates and specialized data	Not directly comparable with USPTO‐50K top‐k exact‐match benchmarks; evaluated using framework‐specific plausibility, pathway‐recovery, or feasibility metrics.
MechRetro [109]	Mechanism‐informed graph transfer	Mechanistic reasoning + uncertainty scoring	Chemically reflective routes; interpretable	Limited benchmark validation serves as the foundational framework later extended by RetroExplainer	Not directly comparable with USPTO‐50K top‐k exact‐match benchmarks; evaluated using framework‐specific plausibility, pathway‐recovery, or feasibility metrics.
RetroExplainer [108]	Multi‐sense graph transformer	Molecular assembly + contrastive learning	High interpretability; multi‐step plausible routes	May not generalize outside biases of training dataset; builds upon and extends the MechRetro framework with improved architectural design	Not directly comparable with USPTO‐50K top‐k exact‐match benchmarks; evaluated using framework‐specific plausibility, pathway‐recovery, or feasibility metrics.
Graph2Edits [113]	Semi‐template graph editing	Sequential graph edits with interpretable edit operations	High top‐1 and round‐trip accuracy; diversity	Requires graph structures and may need more mechanistic insight	USPTO‐50K exact‐match accuracy: 55.1% top‐1 when reaction class is unknown; 67.1% top‐1 when reaction class is provided. Round‐trip accuracy was reported separately at ~86% and is not directly comparable with top‐k exact‐match accuracy.
RetroGFN (GFlowNet) [114]	GFlowNet‐based generation	Feasibility‐weighted sampling	Diverse and feasible routes; strong round‐trip	Lower top‐1 without training adjustment	Not directly comparable with USPTO‐50K top‐k exact‐match benchmarks; evaluated using framework‐specific plausibility, pathway‐recovery, or feasibility metrics.

^a^ Reported values are dataset‐ and protocol‐dependent. For USPTO‐50K exact‐match benchmarks, the table specifies whether reaction class information was provided when this information is available in the cited source. Forward reaction prediction, round‐trip accuracy, MaxFrag accuracy, pathway‐recovery rates, literature‐match rates, and feasibility metrics are not directly comparable with top‐k retrosynthesis exact‐match accuracy and are therefore explicitly distinguished.

### 4.3 Multi‐Modal Explainability and Evaluation Metrics

Current strategies for interpretable retrosynthesis emphasize that no single explanation layer sufficiently captures mechanistic causality. Methods such as SHapley Additive exPlanations (SHAP) [115] have been adapted in recent studies to provide atom‐ and descriptor‐level attributions in reaction and retrosynthesis prediction models. For example, Lu and Zhang applied SHAP [115] to a multitask T5Chem model to interpret sequence‐based reaction predictions at the functional group level, enabling identification of the chemical features driving model outputs across tasks including single‐step retrosynthesis [116]. Such approaches improve interpretability by linking model predictions to chemically meaningful substructures rather than relying solely on confidence scores, while graph‐specific explainers like GNNExplainer localize reactive substructures influencing predictions [117].

Symbolic rule extraction links AI disconnections to documented literature precedents, improving auditability and reproducibility [118]. Because explanations without uncertainty estimates can mislead experimental prioritization, best practice involves presenting mechanistic evidence, confidence intervals, and template support side‐by‐side, prioritizing routes with low execution risk.

Most learning‐based retrosynthesis tools still optimize for top‐k accuracy, which favors dataset performance over laboratory feasibility, creating an incentive misalignment. Key limitations include: (1) Lack of explicit condition prediction (solvents, catalysts, temperature) [108]; (2) Training biases toward common or historically favored reaction classes; and (3) Metrics such as top‐k accuracy failing to capture chemical feasibility [119]. For instance, a high‐accuracy disconnection may still require unscalable catalysts or unsafe solvents, or produce unstable intermediates. To mitigate this, recent initiatives introduce round‐trip verification [18, 120], safety and cost‐aware scoring, and route executability metrics (e.g., Syntheseus benchmarking) [121]. The RetroGFN framework, leveraging feasibility‐weighted GFlowNets, improves route diversity and execution confidence [114], thereby strengthening interpretability while maintaining balanced accuracy.

### 4.4 Toward Trustworthy, Auditable, and Regulatory‐Ready Retrosynthesis

Hybrid strategies combining data‐driven outputs with symbolic or physics‐informed validation are increasingly recognized as essential. For instance, DFT‐augmented filtering enhances assessment of intermediate stability and prevents the ranking of chemically implausible disconnections [122]. However, such workflows increase computational cost, and standardized validation protocols are still lacking. To support experimental adoption, models should rank pathways not only by computational score but also by predicted executability, providing explicit mechanistic rationales and transparent uncertainty estimates.

In summary, interpretable retrosynthesis must incorporate chemically grounded representations, hybrid predictive‐feasibility architectures, multi‐modal explainability, and decision metrics tied to experimental execution and safety. Authors should report chemist‐relevant performance metrics, validate key predictions experimentally, and document dataset biases and failure modes. Only models that demonstrate reliable performance in prospective, real‐world settings are likely to achieve broad adoption in the pharmaceutical industry. Ultimately, transparent retrosynthesis systems will underpin regulatory acceptance of AI‐enabled manufacturing routes by enabling auditability, quality‐by‐design principles, and compliance with emerging digital chemistry guidelines in drug development.

## 5 AI‐Enabled Sustainable Synthesis and Green Chemistry Strategies

The integration of AI‐driven retrosynthesis, automated optimization, and sustainability‐aware decision‐making is progressively reshaping medicinal chemistry workflows toward greener and more resource‐efficient synthesis paradigms. Figure 5 summarizes this integrated framework, illustrating how retrosynthesis planning, sustainability metrics, reaction optimization, and closed‐loop experimental feedback converge within AI‐enabled green chemistry workflows.

**Figure 5:**
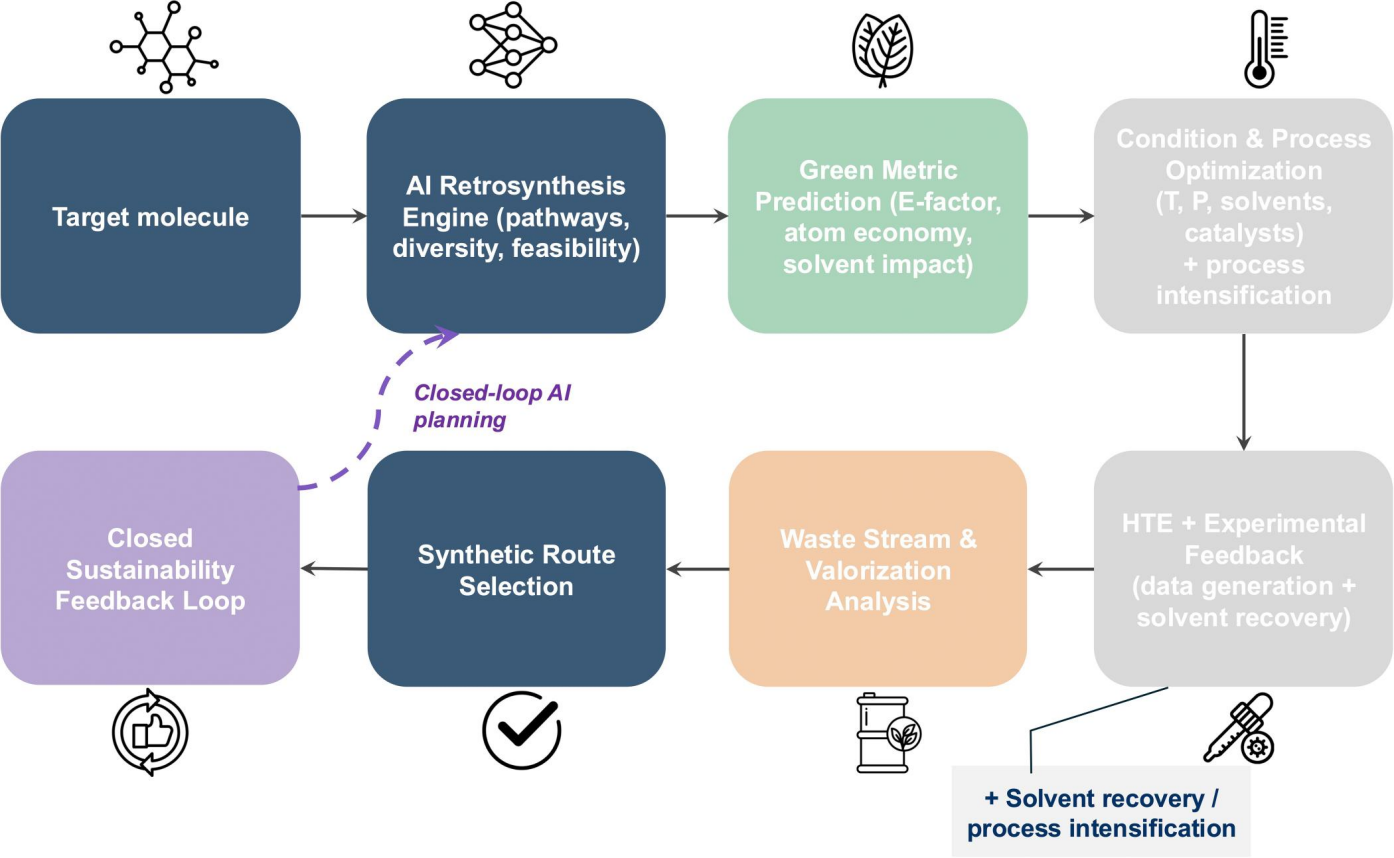
Workflow for AI‐enabled green route design in medicinal chemistry, presented as an overview of the integrated sustainability‐driven synthesis framework, illustrating the integration of retrosynthesis planning, sustainability metrics, and waste minimization within a closed‐loop optimization framework. [Color figure can be viewed at wileyonlinelibrary.com]

### 5.1 From Efficiency to Sustainability in Synthetic Design

Beyond individual reaction optimization, AI is increasingly influencing how medicinal chemistry pipelines are designed by embedding sustainability metrics such as environmental, economic, and operational impact directly into synthetic route planning. This paradigm shift reflects growing regulatory pressure, heightened environmental awareness, and the need for agile, low‐waste discovery workflows.

AI models increasingly integrate green chemistry indicators (e.g., E‐factor, atom economy, solvent greenness) to forecast environmental burden and guide sustainable route planning, enabling chemists to compare alternative pathways before experimental validation [123, 124]. For instance, random forest classifiers capable of estimating E‐factors have been validated industrially for forecasting waste generation and atom economy, providing actionable insights before experimental execution [123]. Together, these developments are transforming green chemistry from a post hoc evaluation into an active design parameter within AI‐assisted synthesis planning.

### 5.2 Toward Autonomous Green Chemistry and Circular Workflows

AI‐driven sustainability is progressing toward autonomous, circular synthesis through the fusion of predictive environmental screening, dynamic process control, and SDLs. Real‐time analytics and flow chemistry allow continuous adaptation of conditions to minimize energy and material waste.

#### 5.2.1 Autonomous Green Chemistry

Aligned with circular economy principles, early‐stage computational tools are being explored to theoretically model waste‐stream compositions and suggest potential recyclability opportunities. While still in their infancy, integrating these models with life‐cycle assessment data may eventually enable the proactive identification of lower‐impact reagents, catalysts, and solvents. Broader applications within pharmaceutical research and development also demonstrate AI's potential in predictive maintenance, intelligent inventory management, and recycling strategies, ultimately enhancing resource efficiency and regulatory compliance [125].

#### 5.2.2 Circular and Chemist‐in‐the‐Loop Frameworks

Critically, sustainable automation depends on chemist‐in‐the‐loop systems, which combine computational prediction with experimental intuition and evolving regulatory standards. Platforms such as AI4Green exemplify this integration, offering transparent solvent selection, energy optimization, and recovery strategies with full data auditability [63].

Embedding AI in sustainable synthesis therefore represents a strategic inflection point, moving beyond traditional metrics of yield and cost toward adaptive, transparent, and environmentally resilient chemistry [125].

## 6 Challenges and Limitations of AI in Synthesis

### 6.1 Data Quality and Availability

While AI tools have demonstrated significant promise in synthetic chemistry, their performance remains constrained by the quality, completeness, and representativeness of available data. Reaction datasets are often noisy, incomplete, and biased, reducing model robustness and generalizability [126]. Moreover, model collapse (the degradation that occurs when models train on their own generated data) can compromise novelty and diversity. Shumailov et al. [127] showed that generative LLMs and variational autoencoders gradually lose rare reaction types over successive training rounds, emphasizing the need for curated, diverse, and experimentally validated datasets. Improving data transparency and adopting open reaction standards (e.g., ORD) will be key to preventing generative collapse and ensuring model reproducibility.

### 6.2 Chemical Complexity and Model Limitations

Despite remarkable advances, AI models still face intrinsic difficulties in predicting stereochemical and mechanistic outcomes, particularly under multi‐step or flow conditions. Most ML models trained on coarse experimental descriptors lack quantum‐chemical precision at an affordable computational cost. Quantum machine learning (QML) offers conceptual promise, but current quantum hardware remains immature and unsuitable for routine synthetic planning.

Transformer‐based LLMs, while transformative in NLP, lack intrinsic chemical structure awareness. Decoder‐only models (e.g., GPT‐4, Gemini) are limited in their capacity to encode reaction context, whereas encoder‐based models (e.g., BERT, ChemBERTa [37]) help in reaction prediction but cannot fully capture multistep logic or three‐dimensional (3D) conformational nuances. Domain‐specific systems like ChemCrow [4], BatGPT‐Chem may yield syntactically valid yet chemically implausible SMILES, reinforcing their current role as assistive tools rather than autonomous chemists [128]. Multimodal models integrating text and visual data struggle with spectral interpretation and 3D reconstruction from NMR, limiting mechanistic analyses [129, 130].

### 6.3 Translational Challenges: Integration, Scalability, and Reproducibility

While robotic and autonomous synthesis platforms enable HTE and closed‐loop optimization, their large‐scale adoption remains constrained by multiple interconnected barriers that limit real‐world deployment and validation of AI‐driven synthesis workflows. In practice, these systems still operate within narrow chemical domains and require manual oversight for complex reaction classes [131]. Benchmarking indicates a persistent disconnect between digital retrosynthetic feasibility and multi‐step laboratory execution, with template‐free models often proposing implausible or infeasible pathways. Furthermore, the lack of standardized machine‐readable execution languages (e.g., XDL) and limited cross‐platform interoperability severely constrain automation scalability. Future progress will depend on the real‐time fusion of sensor feedback, adaptive learning, and robotic control, enabling the context‐aware decision‐making necessary for reliable autonomous synthesis.

Compounding these technical integration hurdles, economic and scalability constraints further limit the translation of these systems into routine pharmaceutical practice. Automated and continuous pharmaceutical manufacturing requires substantial upfront capital and integration costs, with the return on investment strongly dependent on production scale [132]. As Casas‐Orozco et al. [78] demonstrated, continuous manufacturing reduces per‐unit costs for high‐volume programs but offers only marginal advantages for small exploratory projects, limiting its adoption in early‐stage medicinal chemistry. Transitioning from small‐scale automated systems to industrial‐scale operations introduces further challenges regarding reaction yield, safety, and cost efficiency, which current AI–robotic platforms are not fully equipped to manage alone [99]. Hybrid digital–experimental models and modular flow systems could mitigate these limitations by enabling scalable, on‐demand synthesis.

Ultimately, overcoming these practical barriers requires establishing rigorous reproducibility and trust. DL models are highly sensitive to changes in data curation, software dependencies, and hardware, often producing non‐replicable results across laboratories. Their black‐box nature undermines interpretability, making XAI approaches essential. Addressing these challenges requires progress across multiple fronts: chemically informed model architectures, seamless integration with automated experimentation, and standardized interpretable frameworks. Embedding XAI layers and establishing community benchmarks are critical steps toward building reproducibility and trust, transforming AI from a purely predictive tool into a reliable, collaborative partner in molecular discovery. Ultimately, overcoming these challenges is essential to enable robust, scalable, and experimentally validated deployment of AI‐driven synthesis in real‐world pharmaceutical settings.

### 6.4 Critical Appraisal: Achievements, Proofs‐of‐Concept, and Future Expectations

Building on the comparative analysis and limitations discussed in previous sections, to critically assess the current landscape of AI‐driven synthesis, it is essential to distinguish between demonstrated industrial capabilities, academic proofs‐of‐concept, and future expectations, thereby enabling a structured interpretation of current progress and remaining limitations. At present, AI has achieved robust performance in well‐defined and data‐rich chemical domains. Predictive models for reaction outcomes, retrosynthesis planning for known scaffolds, and BO for reaction conditions are increasingly integrated into pharmaceutical workflows, where they contribute to measurable gains in efficiency and resource utilization. AI‐assisted retrosynthesis platforms and industrial route‐design systems have demonstrated practical utility in streamlining route design for known chemical scaffolds, particularly in industrial contexts. However, these successes remain context‐dependent and are often limited to constrained chemical spaces. Performance frequently degrades for highly novel, stereochemically complex, or out‐of‐distribution targets [39], where data scarcity and mechanistic uncertainty remain limiting factors.

In contrast, the integration of AI with automated experimental platforms, including SDLs [98] and robotic chemists [53], largely remains at the proof‐of‐concept stage. While recent studies demonstrate autonomous multi‐step synthesis and closed‐loop optimization, these systems are typically restricted to standardized reaction classes and require highly controlled environments, limiting their immediate applicability to broader medicinal chemistry practice. These platforms also rely on substantial hardware customization and are not yet capable of handling heterogeneous reactions or complex workup procedures common in exploratory synthesis.

Looking forward, the field increasingly aims toward fully autonomous, self‐optimizing discovery pipelines that integrate synthesis planning, experimental execution, and sustainability metrics. However, realizing this vision requires overcoming major challenges, including data standardization, robustness across out‐of‐distribution chemistry, and the incorporation of manufacturability, environmental constraints, and regulatory considerations into AI‐driven decision‐making. In particular, addressing the lack of standardized, machine‐readable experimental data and improving the capture of negative results remain critical bottlenecks.

Overall, a balanced perspective indicates that AI‐driven synthesis is best understood not as a replacement for chemists, but as an augmentative framework, where computational scalability and automation are guided by human expertise to ensure chemical validity, reproducibility, and practical relevance. From our perspective, the near‐term impact of AI in medicinal chemistry will not be defined by full automation, but by the effective integration of complementary AI methodologies within human‐guided workflows that prioritize chemical plausibility, reproducibility, and scalability.

## 7 Conclusion and Future Outlook

AI has contributed to measurable progress in reaction prediction, retrosynthesis, and laboratory automation, although its broader impact remains dependent on data quality, experimental validation, and integration with real‐world workflows. From an expert perspective, three major trends are currently shaping the field: the transition from isolated predictive models to integrated synthesis workflows, the emergence of LLM‐driven multi‐modal reasoning systems, and the increasing convergence of computational design with automated experimentation. Future progress will require combining deeper physical modeling with human–AI collaboration and improved understanding of chemical reactivity. QML holds conceptual promise [133], but routine application is limited by hardware constraints and signal noise. Classical hybrid strategies, combining ML with quantum‐chemical descriptors such as DFT‐augmented features, currently offer the best balance between accuracy and computational cost. In the short term, focusing on experimentally validated hybrid models is pragmatic, while QML matures alongside more robust hardware.

LLMs are being explored as interactive synthesis‐support tools, incorporating reaction conditions, spectral patterns, and mechanistic cues to assist experimental chemists. Systems such as ChemCrow [4] and Auto‐GPT derivatives integrate cheminformatics with literature mining to support retrosynthetic design. These models enhance creativity under uncertainty but remain collaborative aids rather than autonomous chemists. A realistic assessment of the field suggests that, while AI has demonstrated clear value in accelerating specific stages of synthesis and design, its broader impact is still constrained by data limitations, lack of standardization, and incomplete experimental validation across diverse chemical spaces. Future iterations may enable tighter integration with automated laboratories for closed‐loop experimentation, although such capabilities are still under active development and require further validation across diverse chemical domains.

Building community trust will require open benchmarks, standardized evaluation protocols, and transparent datasets. Initiatives such as Syntheseus [121] and SciHorizon [134], alongside ORD [62] and CASP‐style retrosynthesis competitions, help stress‐test AI across unexplored chemical spaces.

Operating in low‐data regimes via transfer learning, few‐shot modeling, and generative representations extends AI utility to rare targets, macrocycles, and natural products, which is critical for early‐stage drug discovery.

The convergence of AI, computational chemistry, and flow‐based experimentation is shifting synthesis from reactive troubleshooting to proactive, data‐driven exploration. Sustainability‐oriented tools such as AI4Green [63] embed environmental and operational metrics in route planning. Importantly, these innovations reinforce a model of AI‐augmented medicinal chemistry in which computational capabilities complement, rather than replace, human expertise.

As discussed in Section 6.4, distinguishing between current capabilities, proof‐of‐concept systems, and future expectations is essential for a realistic assessment of the field. To realize AI's full potential, three key enabling elements must be established: (1) Reproducibility through open science; (2) modular and transparent AI frameworks; and (3) strong feedback loops between computation and experiment. These elements collectively define a realistic pathway toward scalable and trustworthy AI‐driven synthesis. With these elements, AI has the potential to improve synthetic efficiency and sustainability, provided that its limitations are addressed through careful validation and integration with experimental workflows. Ultimately, the next frontier lies in the integration of AI‐driven reasoning with autonomous experimentation under sustainable constraints. Achieving this vision will require rigorous validation and effective integration with experimental workflows.

## Conflicts of Interest

The authors declare no conflicts of interest.

## Data Availability

Data sharing is not applicable as no new data have been generated.
